# Exercise training reduces oxidative damage in skeletal muscle of septic rats

**DOI:** 10.1186/cc10150

**Published:** 2011-06-22

**Authors:** CW Coelho, PR Jannig, AB Souza, H Fronza, GA Westphal, F Petronilho, PM Silva, F Dal-Pizzol, E Silva

**Affiliations:** 1 Programa de Pós-Graduação da FMUSP, São Paulo - SP, Brazil; 2 UNIVILLE, Joinville - SC, Brazil; 3 UDESC, Joinville - SC, Brazil; 4 CEDAP, Joinville - SC, Brazil; 5 Laboratorio de Fisiopatologia Experimental, UNESC, Criciuma - SC, Brazil; 6 Hospital Israelita Albert Einstein, São Paulo - SP, Brazil

## Introduction

Septic patients frequently develop critical illness myopathies (CIMs) that may represent a crucial factor for prolonged intensive care unit treatment and for ventilator weaning delay. Experimental findings have identified that oxidative stress plays a role in causing muscle depletion in chronic pathological states like sepsis. It is well documented that regular moderate physical exercise can decreased oxidative stress and enhance antioxidant functions.

## Objective

To investigate whether exercise training reduces oxidative damage in septic rats induced by cecal ligation and perforation (CLP).

## Methods

Wistar rats were randomly assigned to three groups: Sham (submitted to a fake surgery), CLP, and CLP that was previously trained (CLPT). The exercise training protocol consisted of 8 weeks of running on a treadmill, 5 days/week, for 60 minutes at 60% of the maximal running speed obtained on the graded treadmill test. Rats were subjected to CLP surgery; after 120 hours of surgical procedure they were killed by decapitation. Oxidative damage of lipids (thiobarbituric acid reactive species (TBARS)) and proteins (carbonyl groups) were analyzed in Soleus (type I fiber) and plantaris (type II fiber) muscles.

## Results

See Table 1

**Table 1:** Levels of TBARS and carbonyl of soleus and plantaris muscles

Analysis	Muscle	Sham	CLP	CLPT
TBARS (nmol/mg protein × 10^4^)	Soleus	43.0 ± 5.4 (11)	60.2 ± 5.9 (13)*	39.7 ± 5.5 (6)**
	Plantaris	31.3 ± 2.6 (10)	55.3 ± 7.3 (11)*	27.8 ± 5.6 (5)**
Carbonyl (nmol/mg protein × 10^12^)	Soleus	38.8 ± 4.3 (11)	50.9 ± 4.6 (12)^†^	31.3 ± 4.6 (6)**
	Plantaris	28.8 ± 3.9 (10)	49.7 ± 5.1 (13)*	45.5 ± 6.9 (6)^†^

Values presented as mean ± SEM. *P < 0.05 vs. sham. **P < 0.05 vs. CLP. ^†^*P *= 0.06 vs. sham.

## Conclusion

**Figure 1:**
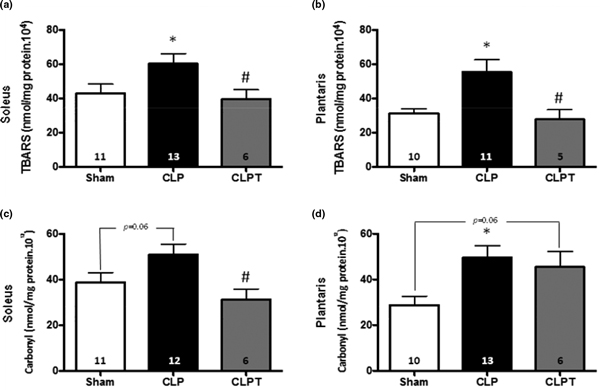
**Levels of TBARS of soleus (a) and plantaris (b); and levels of protein carbonyl of soleus (c) and plantaris (d)**. Values presented as mean ± SEM. *Significant difference in relation to Sham group (*P *< 0.05); ^#^Significant difference in relation to CLP group (*P *< 0.05).

TBARS and carbonyl analysis for CLPT are lower than for CLP with statistical significance, except for carbonyl plantaris with *P *= 0.06 (Table 1 and Figure 1). Our data supported that exercise training before sepsis could decrease oxidative damage in both muscle fiber types.

